# EHMTI-0129. 3D-cinema and headache: preliminary results of a prospective cross-sectional study

**DOI:** 10.1186/1129-2377-15-S1-C8

**Published:** 2014-09-18

**Authors:** M Braschinsky, A Rakitin, N Zmachinskaja, O Zukovskaja, B Saar, A Karask, L Sabre

**Affiliations:** 1 Neurology Clinic, Tartu University Hospital, Tartu, Estonia; 2 Faculty of Medicine, Tartu University, Tartu, Estonia

## Introduction

3D-cinema is a growing audio-visual experience world-wide. There are anecdotal reports, that the exposure to 3D-movies can induce headache.

## Aim

To establish the frequency of occurrence of headache after viewing 3D-movies in commercial cinemas, to describe the headache characteristics in response to the exposure and to assess possible contributing co-factors.

## Methods

Specifically designed questionnaire, containing demographic data, name of a movie, prior history of headaches, exposure to possible contributing co-factors and headache descriptors, was distributed to 6000 visitors of three major cinemas in Estonia.

## Results

By the date of the abstract's submission 1131 persons (337 men and 794 women) have responded to the questionnaire. The mean age of responders was 33 years (6-74). Among them 26.6 % reported to have had a headache in response to work in front of a non-3D computer screen, 13.9% in relation to 3D movies, and only 1.5% after watching 2D-films earlier in life. In this study, however, 6.2% of men and 11.6% of women reported to have headache: women were at a higher risk of occurrence of headache after or during the 3D-cinema compared to men (OR=1.78; 95%CI=1.18-2.69). More specific and final results, including the subgroups analysis will be presented.

## Conclusions

Watching 3D-movies at a cinema can cause a headache. Further studies are needed to confirm the possible risk factors of individual susceptibility to the 3D-cinema headache. There is a need of rising public awareness of possible discomfort people may suffer during and after the explosion to 3D-movies.

No conflict of interest.

